# Pattern of distant metastases in primary extrahepatic bile‐duct cancer: A SEER‐based study

**DOI:** 10.1002/cam4.1772

**Published:** 2018-09-12

**Authors:** Xuan Wang, Guang‐Yang Yu, Mo Chen, Ran Wei, Jinhong Chen, Zheng Wang

**Affiliations:** ^1^ Department of General Surgery Huashan Hospital Fudan University Shanghai China; ^2^ Cancer Metastasis Institute Fudan University Shanghai China; ^3^ Institutes of Biomedical Sciences Fudan University Shanghai China

**Keywords:** extrahepatic cholangiocarcinoma, gallbladder cancer, metastasis, SEER

## Abstract

Extrahepatic bile duct cancer (EBDC) is a combined type of malignancy mainly consisting of extrahepatic cholangiocarcinoma and gallbladder cancer. Clinically, it is featured with latent symptoms and early metastasis, leading to a poor prognosis. Therefore, this cohort study aimed to depict the possible metastatic patterns of EBDC of diverse sub‐types and evaluate the prognostic significance of diverse metastatic destinations with data from the clinical database. Relevant data of total 4061 confirmed EBDC patients diagnosed between 2010 and 2013 from the Surveillance, Epidemiology and End Results (SEER) database was obtained. We applied *t* test to describe the baseline data of patients included and used chi‐square test to compare the distribution of distant metastatic sites. We further adopted odds ratio assess the combined metastatic patterns and compared survival difference of patients with different distal metastasis organ by Kaplan‐Meier analysis**.** We identified totally 4061 patients over 18 years old diagnosed with extrahepatic bile tract malignancies between 2010 and 2013, with clear metastatic status and follow‐up data, without primary malignancies. Liver and distant lymph (DL) are the two most common sites as a single metastasis organ. In combined metastasis patterns, bi‐organ is more frequent than the other types. Lung is the organ preferentially for bi‐organ metastasis, while bone and distant lymph similarly intend to co‐metastasize with brain. Distal metastasis in EBDC patients indicates an extremely poor prognosis. According to the final analysis results, malignancies in extrahepatic bile duct exhibit similar metastatic patterns, suggesting that we can regard them as a unity to assess its development. Profound differences exist in distribution of distant extrahepatic metastatic sites and their combinations. Results from our studies would provide some information for follow‐up strategies and future studies.

## 1 INTRODUCTION

Extrahepatic bile duct cancer (EBDC), as a large group of malignancies, consists of cancer in perihilar and distal extrahepatic bile duct (extrahepatic cholangiocarcinoma, EC) as well as in the gallbladder (gallbladder cancer, GC).^1^, ^2^ Compared with its counterpart occupied in the liver (roughly 5%‐10% of total cholangiocarcinomas), which is currently classified as primary liver cancer.^3^, ^4^ EBDC is unique not only from the perspective of anatomy, but also from epidemiology, pathogenesis and its further development compared with other malignancies.^5^, ^6^ Over 12 000 cases of extrahepatic biliary tract cancers are diagnosed annually in the United States, more than 60% are gallbladder cancers. The rest, approximately 3000 cases per year, are extrahepatic cholangiocarcinomas.^7^ Although the incidence seems to remain stable currently in the USA, this type of cancer can initially be symptom‐free and diagnosed at a relatively late stage. Thus, when cases are confirmed, most of them are locally and distally advanced which can be extremely lethal.^8^

By far, surgical resection remains to be the only potentially curative treatment for EBDC.^9^ Still, patients receiving curative surgical resection end up with disappointing prognosis. Previous studies stated that 5‐year survival rates vary between 12% and 54% in spite of radical surgeries.^10^, ^11^ This frustrating prognosis is mainly attributed to the early distal metastasis and local recurrence.^12^ Perihilar EBDC is characterized by intrahepatic ductal extension, making liver, the adjacent organ as a common site for early metastasis.^13^ Although surrounding structure can be primarily spread, distant metastasis occurring late in the course of these diseases are most often found in the liver, lung, and peritoneum, which are similar in both perihilar and distal EBDC.^14^ Yet the pattern of EBDC metastasis still requires further investigation. Also whether diverse metastatic destinations are consistent with different prognosis needs solid clinical evidence. We aimed to elaborate the correlation between different metastatic lesions and prognosis of EBDC patients, using data extracted from the SEER database. And we wished to conclude the basic pattern of EBDC distal metastases.

## 2 METHODS

### 2.1 Cohort population

**Figure 1:**
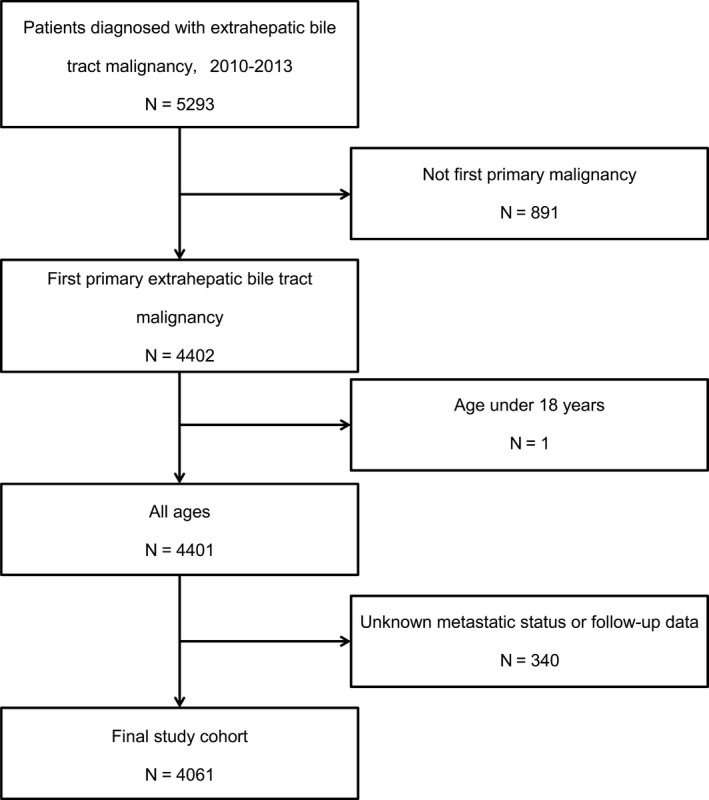
Flowchart of patient inclusion in this cohort study

We established the cohort study using data extracted from the Surveillance, Epidemiology and End Results (SEER) national database. The detailed cohort selection procedure was summarized (Figure 1). In short, we identified totally 4061 patients over 18 years old diagnosed with extrahepatic bile tract malignancies between 2010 and 2013, with clear metastatic status and follow‐up data, without primary malignancies. At the very beginning, we intended to include multiple metastatic sites for a brand view. However, the SEER database contains metastatic information only in bone, brain, liver, lung, and distal lymph nodes (DL), which can basically cover extensive metastatic organs for EBDC. Approval was obtained from the Shanghai Huashan hospital Review Board, and a data use agreement was signed for this project.

### 2.2 Statistical analysis

Demographic and clinical characteristic analysis of populations were performed by descriptive statistics. We analyzed frequency distribution among different metastatic organs, comparing them by the odds ratio calculation. Overall survival and cancer‐specific survival among different metastatic sites was analyzed with Kaplan‐Meier curves and log‐rank test. Statistical significance was considered at two‐sided *P* value <0.05. The statistical analysis applied statistical software packages Excel (Microsoft Excel, Inc. Microsoft, USA) and SPSS 20.0 (SPSS, Inc., Chicago, IL, USA).

## 3 RESULTS

### 3.1 Patient characteristics

Among the 4061 patients we finally selected from SEER database, 496 cases (12.2%) are perihilar bile duct cholangiocarcinoma, 663 cases (16.3%) are distal bile duct cholangiocarcinoma, and 2902 cases (71.5%) are gallbladder cancer. The clinical baseline characteristic data indicated that basically there was no significant difference in gender, marital status, age, and race among these four metastatic organs (Table 1). Although the race analysis suggested that African‐American patients may have a higher risk for possible liver metastasis, the results may have something to do with social‐economic background. It requires further investigation.

**Table 1:** Clinical baseline characteristics of EBDC patients in SEER database

Characteristic	Brain metastasis		*P* value	Bone metastasis		*P* value	Lung metastasis		*P* value	Liver metastasis		*P* value	DL metastasis		*P* value
	No (%)	Yes (%)		No (%)	Yes (%)		No (%)	Yes (%)		No (%)	Yes (%)		No (%)	Yes (%)	
Gender															
Male	1520 (99.6)	6 (0.4)	0.374	1486 (97.4)	40 (2.6)	0.174	1464 (95.9)	62 (4.1)	0.317	1205 (79.0)	321 (21.0)	0.011	1459 (95.6)	67 (4.4)	0.446
Female	2529 (99.8)	6 (0.2)		2485 (98.0)	50 (2.0)		2415 (95.3)	120 (4.7)		1914 (75.5)	621 (24.5)		2410 (95.1)	125 (4.9)	
Marriage															
Married	2015 (99.8)	4 (0.2)	0.124	1974 (97.8)	45 (2.2)	0.399	1937 (95.9)	82 (4.1)	0.425	1547 (76.6)	472 (23.4)	0.308	1920 (95.1)	99 (4.9)	0.181
Unmarried	1839 (99.7)	6 (0.3)		1807 (97.9)	38 (2.1)		1754 (95.1)	91 (4.9)		1424 (77.2)	421 (22.8)		1766 (95.7)	79 (4.3)	
Unknown	195 (99.0)	2 (1.0)		190 (96.4)	7 (3.6)		188 (95.4)	9 (4.6)		148 (75.1)	49 (24.9)		183 (92.9)	14 (7.1)	
Age															
<60	862(99.3)	6 (0.7)	0.009	843 (97.1)	25 (2.9)	0.054	822 (94.7)	46 (5.3)	0.232	645 (74.3)	223 (25.7)	0.020	813 (93.7)	55 (6.3)	0.010
61‐74	1670 (99.6)	6 (0.4)		1634 (97.5)	42 (2.5)		1611 (96.1)	65 (3.9)		1275 (76.1)	401 (23.9)		1594 (95.1)	82 (4.9)	
≥75	1517 (100.0)	0 (0.0)		1494 (98.5)	23 (1.5)		1446 (95.3)	71 (4.7)		1199 (79.0)	318 (21.0)		1462 (96.4)	55 (3.6)	
Race															
White	3078 (99.7)	10 (0.3)	0.417	3020 (97.8)	68 (2.2)	0.677	2956 (95.7)	132 (4.3)	0.343	2406 (77.9)	682 (22.1)	<0.01	2948 (95.9)	140 (4.5)	0.592
Black	487 (100)	0 (0.0)		478 (98.2)	9 (1.8)		459 (94.3)	28 (5.7)		332 (68.2)	155 (31.8)		461 (94.7)	26 (5.3)	
Others^a^	484 (99.6)	2 (0.4)		473 (97.3)	13 (2.7)		464 (95.5)	22 (4.5)		381 (78.4)	105 (21.6)		460 (94.7)	26 (5.3)	
Grade^b^															
I	384 (100.0)	0 (0.0)	0.076	381 (99.2)	3 (0.8)	<0.01	377 (98.2)	7 (1.8)	<0.01	361 (94.0)	23 (6.0)	<0.01	379 (98.7)	5 (1.3)	<0.01
II	1156 (100.0)	0 (0.0)		1150 (99.5)	6 (0.5)		1133 (98.0)	23 (2.0)		1007 (87.1)	149 (12.9)		1119 (96.8)	37 (3.2)	
III	1063 (99.6)	4 (0.4)		1047 (98.1)	20 (1.9)		1033 (96.8)	34 (3.2)		847 (79.4)	220 (20.6)		1032 (96.7)	35 (3.3)	
IV	43 (100.0)	0 (0.0)		41 (95.3)	2 (4.7)		40 (93.0)	3 (7.0)		32 (74.4)	11 (25.6)		41 (95.3)	2 (4.7)	
Unknown	1403 (99.4)	8 (0.6)		1352 (95.8)	59 (4.2)		1296 (91.8)	115 (8.2)		872 (61.8)	539 (38.2)		1298 (92.0)	113 (8.0)	
Size (cm)															
<2.0	578 (99.8)	1 (0.2)	0.306	569 (98.3)	10 (1.7)	0.122	566 (97.8)	13 (2.2)	<0.01	528 (91.2))	51 (8.8)	<0.01	568 (98.1)	11 (1.9)	<0.01
2.0‐4.9	1137 (99.9)	1 (0.2)		1120 (98.4)	18 (1.6)		1112 (97.7)	26 (2.3)		964 (84.7)	174 (15.3)		1096 (96.3)	42 (3.7)	
=>5.0	611 (99.7)	2 (0.3)		600 (97.9)	13 (2.1)		586 (95.6)	27 (4.4)		448 (73.1)	165 (26.9)		572 (93.3)	41 (6.7)	
Unknown	1723 (99.5)	8 (0.5)		1682 (97.2)	49 (2.8)		1615 (93.3)	116 (6.7)		1179 (68.1)	552 (31.9)		1633 (94.3)	98 (5.7)	
Site															
Perihilar bile duct	495 (99.8)	1 (0.2)	0.652	479 (96.6)	17 (3.4)	0.086	467 (94.2)	29 (5.8)	0.027	381 (76.8)	115 (23.2)	<0.01	474 (95.6)	22 (4.4)	0.58
Distal bile duct	662 (99.8)	1 (0.2)		653 (98.5)	10 (1.5)		645 (97.3)	18 (2.7)		584 (88.1)	79 (11.9)		643 (97.0)	20 (3.0)	
Gallbladder	2892 (99.7)	10 (0.3)		2839 (97.8)	63 (2.2)		2767 (95.3)	135 (4.7)		2154 (74.2)	748 (25.8)		2752 (94.8)	150 (5.2)	
Radiation															
Radiation	471 (99.2)	4 (0.8)	0.200	463 (97.5)	12 (2.5)	0.625	471 (99.2)	4 (0.8)	<0.01	2667 (74.4)	919 (25.6)	<0.01			
No radiation	3578 (99.8)	8 (0.2)		3508 (97.8)	78 (2.2)		3408 (95.0)	178 (5.0)		452 (95.2)	23 (4.8)				

a Others include American Indian/AK Native, Asian/Pacific Islander.

b Grade: I‐Well differentiated; II‐Moderately differentiated; III‐Poorly differentiated; IV‐Undifferentiated.

### 3.2 Metastatic pattern

**Figure 2:**
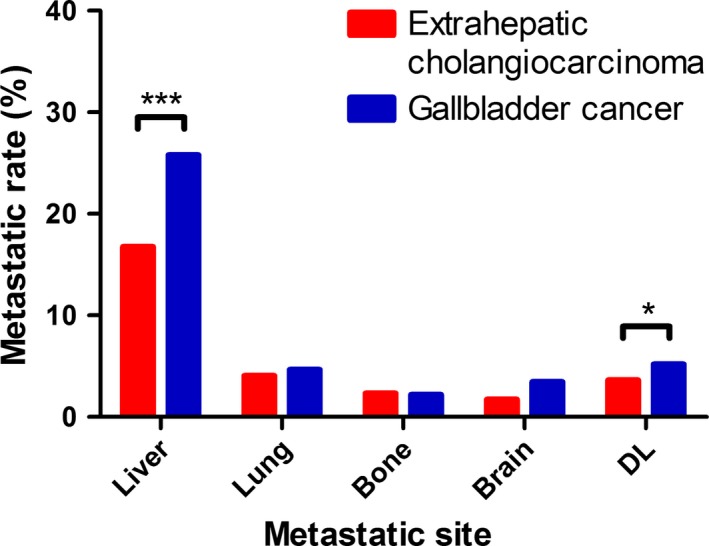
Distribution of distant metastatic sites according to EBDC classification (extrahepatic cholangiocarcinoma and gallbladder cancer). DL, distant lymph. *P < 0.05, ***P < 0.01 (Chi‐square test)

Based on the original tissues of the malignancies, we classified EBC into extrahepatic cholangiocarcinoma and gallbladder cancer for metastatic distribution comparison. It is clearly shown that both types of EBC had significantly higher possibilities to metastasize into liver than the other four sites (EC: 49.82%, GC: 53.10%). Notably, DL metastasis is more commonly seen than lung, bone and brain (EC: 5.96%, GC: 5.56%) as well (Figure 2).

For EBC tumor characteristics, most metastatic organs (except for the brain) exhibited a similar trend that the higher grade poorly differentiated or undifferentiated the tumors were, the more likely for them to metastasize. This is consistent with our conventional view that poorly differentiated tumors tend to metastasize. Also it seems that larger EBC tumors are more likely to metastasize to lung, liver and DL. Different sites of EBC showed no significantly diverse intends to brain, lung and bone metastasis. However, perihilar bile duct cholangiocarcinoma and gallbladder cancer are two types of EBC with a higher likelihood for liver metastasis, while distal duct cholangiocarcinoma may come with a lower likelihood compared with the other two types.

### 3.3 Combination of metastasis

**Figure 3:**
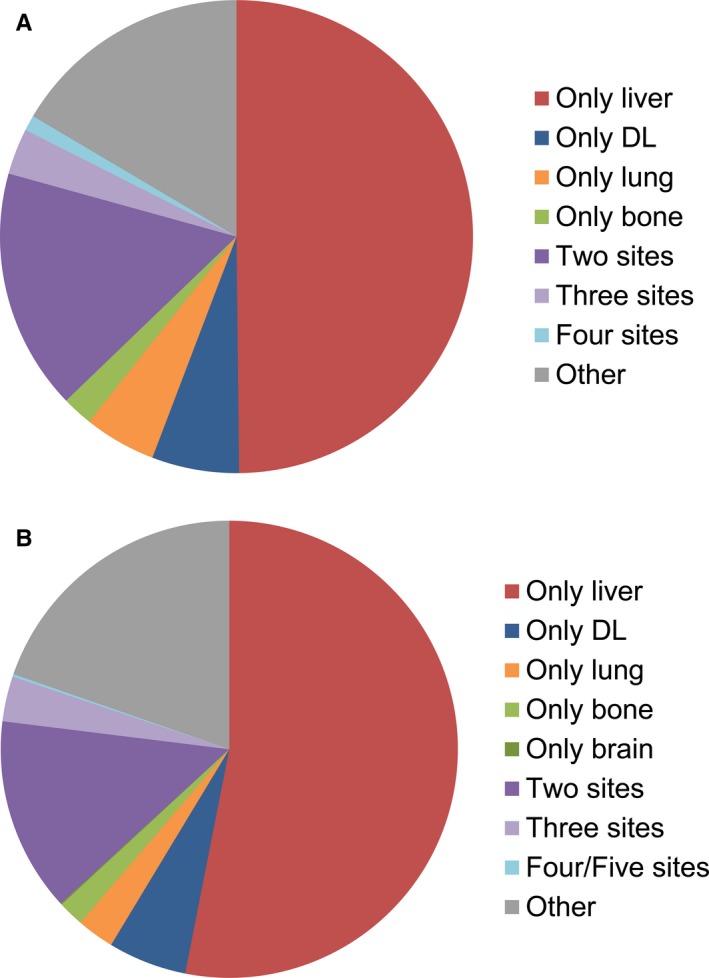
Relative rates of single and combined metastatic sites in extrahepatic cholangiocarcinoma (A) and gallbladder cancer (B). DL, distant lymph

**Figure 4:**
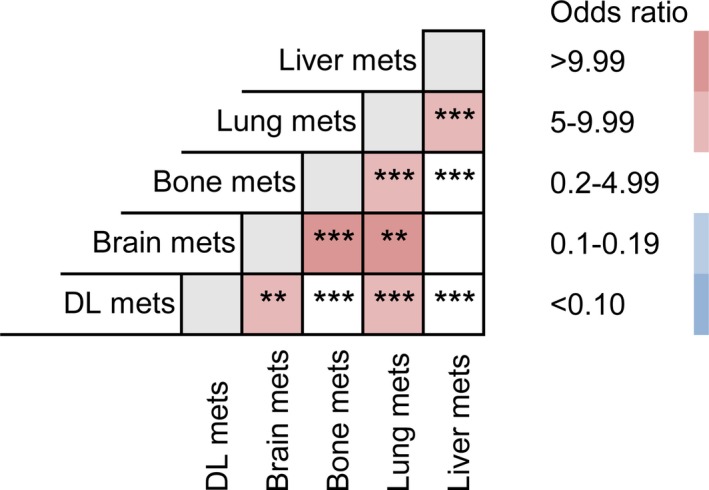
Odds ratio comparison among different metastatic combinations

Many patients are diagnosed with more than one metastatic lesion simultaneously or sequentially. The pie chart showed the proportions of each single metastasis and combined metastasis patterns in both EC and GC patients (Figure 3). As previously stated, liver and DL are two leading metastatic sites as a single metastasis for EBDC. For combined metastasis, bi‐site pattern (EC: 16.49%, GC: 13.75%) is predominantly higher than tri‐site (EC: 3.16%, GC: 3.19%) and tetra‐site pattern (EC: 1.05%, GC: 0.18%). For a better understanding of the specific distribution of bi‐site metastasis, we compared the odds ratio of each possible combination between all five sites (Figure 4). Lung metastasis turned out to be the most common site for bi‐site combined metastasis (ORs of combined metastasis with liver, bone, brain, and DL are 7.918, 6.252, 10.874 and 7.744 separately). Liver metastasis specially correlates with lung metastasis. Their combined co‐metastasis is far more frequent than any other co‐metastasis combined with liver. Bone metastasis also preferentially intends to co‐metastasize with brain besides lung (OR: 9.756), which is similar to the pattern of DL metastasis (OR: 6.808).

### 3.4 Survival

**Figure 5:**
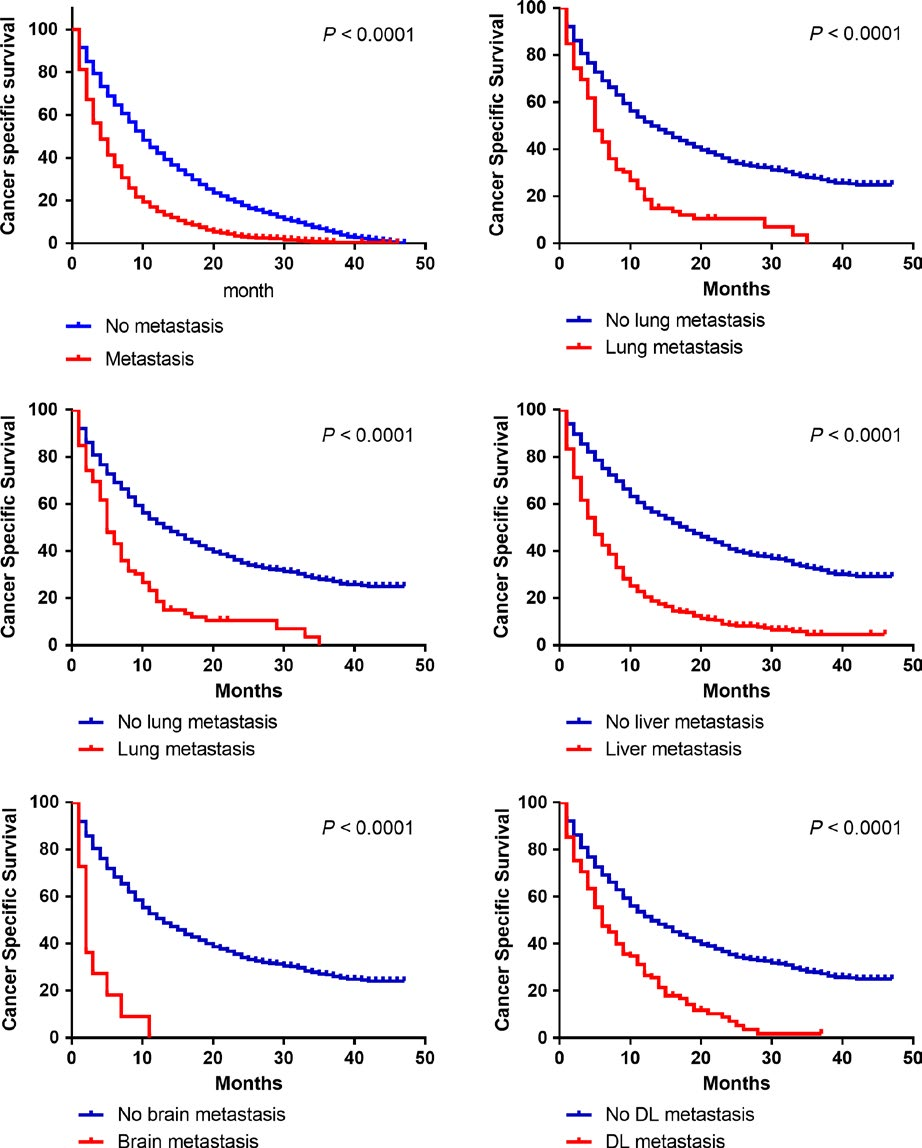
Kaplan‐Meier analysis of cancer specific survival in patients with and without metastasis

**Figure 6:**
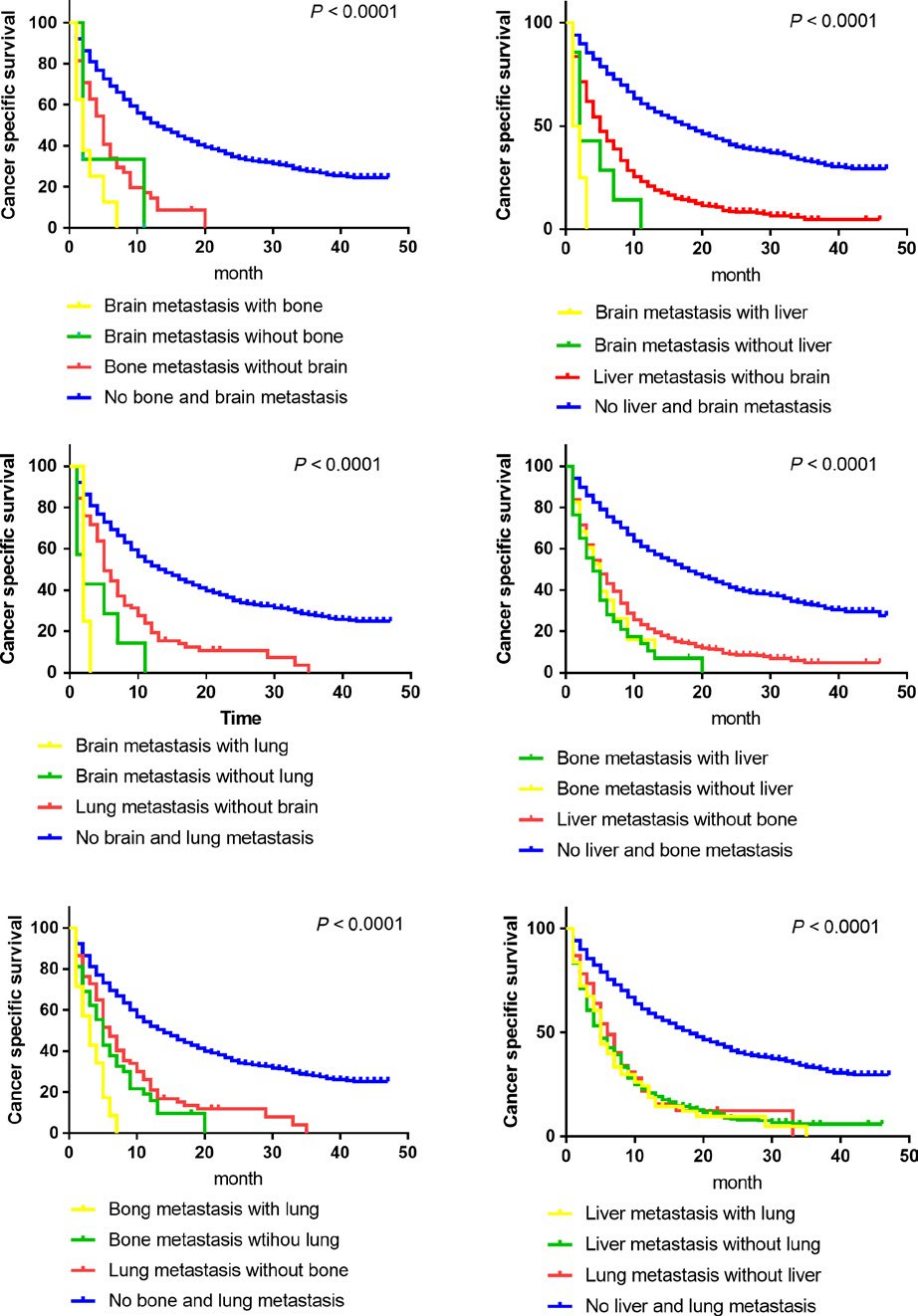
Kaplan‐Meier analysis of cancer‐specific survival comparisons in patients with differed bi‐organ metastasis patterns

In this cohort study, we observed 2558 deaths (63.0%) among 4061 patients and calculated one‐year overall‐survival (OS; Table 2). The one‐year OS differences between patients with or without metastasis in all five sites are extraordinarily (with *P* < 0.0001) large (liver 50.0% vs 15.6%, lung 43.3% vs 12.7%, bone 42.7% vs 9.7%, brain 42.1% vs 0.0%, DL 43.1% vs 20.1%). Quantified survival analysis data of the CSS can be better intuitively seen in the Kaplan‐Meier survival curves (Figure 5). We further compared survival differences between different bi‐organ metastasis patterns among the four solid organs (lung, liver, bone and brain). The survival curves described the varied prognosis of statistical significance (Figure 6). As these figures clearly indicated, combined brain metastasis resulted in worse prognosis than the other organs. And bone combined lung metastasis ended up worse than the separated single metastasis. Surprisingly, like the combination between lung and liver, also between liver and bone, bi‐organ metastasis actually suggested no worse ending that the separated single metastasis.

**Table 2:** Survival analysis in diverse metastatic organs

Parameter	1‐year OS (%)	Univariate analysis	
		Log rank χ^2^ test	*P*
Liver			
No metastasis	50.0	514.882	<0.01
Metastasis	15.6		
Lung			
No metastasis	43.3	96.659	<0.01
Metastasis	12.7		
Bone			
No metastasis	42.7	67.853	<0.01
Metastasis	9.7		
Brain			
No metastasis	42.1	18.644	<0.01
Metastasis	0.0		
Distal lymph nodes			
No metastasis	43.1	62.666	<0.01
Metastasis	20.1		

## 4 DISCUSSION

Extrahepatic bile duct cancer has been known as asymptomatic free in the early phases, thus distal metastasis often occurred when initially diagnosed.^1^ Understanding the metastatic pattern and preferential metastasis distribution will be beneficial to optimal treatment selection and better prognosis. In this cohort study, we mainly clarified the following three points: (a) Identifying the distribution of a single organ distal metastasis of EBC. (b) Concluding the combined metastatic patterns and their respective odds ratio. (c) Analyzing survival rates of different metastatic patterns. It is hoped that these results can be meaningful in a clinical environment.

Conventional belief stated that extrahepatic cholangiocarcinoma and gallbladder cancer intend to invade retrogradely along the bile duct into the liver.^14^ Our study further verified the belief with minor correction that distal duct cholangiocarcinoma may be less associated with liver metastasis in comparison with the other two types (liver metastasis is still the major metastasis organ compared with brain, lung, bone and DL). This may be consistent with the relatively further anatomical position. DL metastasis is one common site for metastasis.

Although perihilar cholangiocarcinoma and distal cholangiocarcinoma may differ in regional invasive pattern,^15^ the former spreads along perineural and periductal lymphatic channels. Hilar and pericholedochal nodes in the hepatoduodenal ligament are most commonly involved.^13^ The latter is the same as exocrine cancer of the pancreatic head (along the common bile duct, common hepatic artery, portal vein, posterior and anterior pancreaticoduodenal nodes and nodes along the right lateral wall of the superior mesenteric artery).^16^ But in a view of distant metastasis, we might be able to treat different types of EBC as a unity for liver, lung and peritoneum (not shown in this study) metastasis, which are common target organs for all three types of EBDC, as data suggested in this study.

Most demographic parameters (age, marital status and gender) seem irrelevant with EBDC metastatic distribution except for race. Our study indicated that African‐American patients had a higher risk of liver metastasis. As a matter of fact, previous studies did demonstrate that patients of different races may show diverse metastatic intends.^17^ Whether this otherness is correlated with genetic background or affected by social‐economic factors requires further investigations.

Combined metastasis of EBDC has not been well focused by researchers by far. Clearly bi‐organ metastasis of EBDC are not randomly combined by any two distal metastatic sites, even though we concluded that liver is the most common metastatic site. Liver actually does not preferentially metastasize with other organs (except for lung) as indicated by the odds ratio comparison (OR 0.2‐4.99). The SEER database does not have the data for the sequence of combined metastasis, so we may be unable to clarify the sequential relationship between lung and other metastatic organs. However, lung is the most common metastatic organ in combined metastasis. This would be of great significance in screening and organ‐targeted treatment for metastatic EBDC patients.

Beyond the described metastatic patterns, we found the distinct prognostic significance in differed combined metastasis, which may be a challenge to our conventional knowledge. Not all spontaneous bi‐organ metastasis have a worse prognosis than a single metastasis. This data‐based study may not be able to come forward with an explanation or hypothesis to this result. It is hoped that this meaningful question could be an inspiration for further explorations.

As far as we are concerned, this SEER‐based study is among the pioneering work beginning to view EBDC as a brand unity and explored its metastatic pattern based on direct clinical data. As a retrograde cohort study, its limitations are obvious. Primarily, all the patients enrolled were diagnosed between 2010 and 2013. Moreover, as we mentioned, metastatic information retrieved from the SEER database is restricted to only five sites. That is to say, we might ignore metastasis in other parts, which are not mentioned in the database (like peritoneum for EBDC). Also, the metastatic data we studied was synchronous metastasis at the time of diagnosis, implying that we do not know its future development. For example, Brain metastasis is usually the late‐stage phenomenon, which may not be shown at the diagnosis. In a word, based on the differences of metastatic patterns in a single organ or combined metastases, we suggest that clinicians could take the primary tumor site or first metastatic site into account when designing diagnostic and treatment algorithms.

To sum up, in this SEER‐based cohort study, we found that EBDC (mainly classified into extrahepatic cholangiocarcinoma and gallbladder cancer) metastasize in a similar pattern among different primary sites. Liver and DL are two common sites for a single‐organ metastasis. African‐American EBDC patients are even at a higher risk for liver metastasis. Combined metastasis in EBDC are most commonly occur with bi‐organ metastases, and lung is the organ preferentially for bi‐organ metastasis, indicating patients with lung metastasis might have a higher risk for metastasis in liver, bone, brain and DL.

## CONFLICT OF INTEREST

None declared.

## References

[1] Bile Duct Cancer (Cholangiocarcinoma) Treatment (PDQ(R)): Health Professional Version, 2002. 26389308

[2] Deoliveira ML , Schulick RD , Nimura Y , et al. New staging system and a registry for perihilar cholangiocarcinoma. Hepatology. 2011;53(4):1363‐1371. 21480336 10.1002/hep.24227

[3] Chi Z , Bhalla A , Saeed O , et al. Mucinous intrahepatic cholangiocarcinoma: a distinct variant. Hum Pathol. 2018;78:131‐137. 29698701 10.1016/j.humpath.2018.04.010

[4] Matsuba T , Qiu D , Kurosawa M , et al. Overview of epidemiology of bile duct and gallbladder cancer focusing on the JACC Study. J Epidemiol. 2005;15(Suppl 2):S150‐S156. 16127227 10.2188/jea.15.S150 PMC8639039

[5] Banales JM , Cardinale V , Carpino G , et al. Expert consensus document: Cholangiocarcinoma: current knowledge and future perspectives consensus statement from the European Network for the Study of Cholangiocarcinoma (ENS‐CCA). Nat Rev Gastroenterol Hepatol. 2016;13(5):261‐280. 27095655 10.1038/nrgastro.2016.51

[6] Ahn DH , Bekaii‐Saab T . Biliary cancer: intrahepatic cholangiocarcinoma vs. extrahepatic cholangiocarcinoma vs. gallbladder cancers: classification and therapeutic implications. J Gastrointest Oncol. 2017;8(2):293‐301. 28480068 10.21037/jgo.2016.10.01 PMC5401855

[7] Siegel RL , Miller KD , Jemal A . Cancer statistics, 2018. CA Cancer J Clin. 2018;68(1):7‐30. 29313949 10.3322/caac.21442

[8] Rizvi S , Gores GJ . Pathogenesis, diagnosis, and management of cholangiocarcinoma. Gastroenterology. 2013;145(6):1215‐1229. 24140396 10.1053/j.gastro.2013.10.013 PMC3862291

[9] Kurahara H , Maemura K , Mataki Y , et al. Indication of extrahepatic bile duct resection for gallbladder cancer. Langenbecks Arch Surg. 2018;403(1):45‐51. 28875312 10.1007/s00423-017-1620-7

[10] Nishio H , Ebata T , Yokoyama Y , Igami T , Sugawara G , Nagino M . Gallbladder cancer involving the extrahepatic bile duct is worthy of resection. Ann Surg. 2011;253(5):953‐960. 21490453 10.1097/SLA.0b013e318216f5f3

[11] Foster JM , Hoshi H , Gibbs JF , et al. Gallbladder cancer: defining the indications for primary radical resection and radical re‐resection. Ann Surg Oncol. 2007;14(2):833‐840. 17103074 10.1245/s10434-006-9097-6

[12] Kurahara H , Maemura K , Mataki Y , et al. Relationship between the surgical margin status, prognosis, and recurrence in extrahepatic bile duct cancer patients. Langenbecks Arch Surg. 2017;402(1):87‐93. 27491729 10.1007/s00423-016-1491-3

[13] Nath MC , Torbenson MS , Erickson LA . Perihilar Cholangiocarcinoma. Mayo Clin Proc. 2018;93(3):397‐398. 29502573 10.1016/j.mayocp.2018.01.017

[14] Katayose Y , Nakagawa K , Yamamoto K , et al. Lymph nodes metastasis is a risk factor for bone metastasis from extrahepatic cholangiocarcinoma. Hepatogastroenterology. 2012;59(118):1758‐1760. 22366495 10.5754/hge11806

[15] Ercolani G , Dazzi A , Giovinazzo F , et al. Intrahepatic, peri‐hilar and distal cholangiocarcinoma: three different locations of the same tumor or three different tumors? Eur J Surg Oncol. 2015;41(9):1162‐1169. 26095704 10.1016/j.ejso.2015.05.013

[16] Nagorney DM PTCY . AJCC cancer staging manual, 8th, Amin MB (Ed). Chicago; 2017: 217.

[17] Ikoma N , Blum M , Chiang YJ , et al. Race is a risk for lymph node metastasis in patients with gastric cancer. Ann Surg Oncol. 2017;24(4):960‐965. 27778127 10.1245/s10434-016-5645-x

